# A Predominantly Neolithic Origin for European Paternal Lineages

**DOI:** 10.1371/journal.pbio.1000285

**Published:** 2010-01-19

**Authors:** Patricia Balaresque, Georgina R. Bowden, Susan M. Adams, Ho-Yee Leung, Turi E. King, Zoë H. Rosser, Jane Goodwin, Jean-Paul Moisan, Christelle Richard, Ann Millward, Andrew G. Demaine, Guido Barbujani, Carlo Previderè, Ian J. Wilson, Chris Tyler-Smith, Mark A. Jobling

**Affiliations:** 1 Department of Genetics, University of Leicester, Leicester, United Kingdom; 2 Ty Celyn, Maeshafod, Blaina, Gwent, United Kingdom; 3 Laboratoire d'Etude du Polymorphisme de l'ADN, Faculté de Médecine, Nantes, France; 4 Molecular Medicine Research Group, Peninsula Medical School, Universities of Exeter and Plymouth, Plymouth, United Kingdom; 5 Dipartimento di Biologia ed Evoluzione, Università di Ferrara, Ferrara, Italy; 6 Dipartimento di Medicina Legale e Sanità Pubblica, Università di Pavia, Pavia, Italy; 7 Institute of Human Genetics, Newcastle University, Newcastle upon Tyne, United Kingdom; 8 The Wellcome Trust Sanger Institute, Wellcome Trust Genome Campus, Hinxton, United Kingdom; Massey University, New Zealand

## Abstract

The relative contributions to modern European populations of Paleolithic hunter-gatherers and Neolithic farmers from the Near East have been intensely debated. Haplogroup R1b1b2 (R-M269) is the commonest European Y-chromosomal lineage, increasing in frequency from east to west, and carried by 110 million European men. Previous studies suggested a Paleolithic origin, but here we show that the geographical distribution of its microsatellite diversity is best explained by spread from a single source in the Near East via Anatolia during the Neolithic. Taken with evidence on the origins of other haplogroups, this indicates that most European Y chromosomes originate in the Neolithic expansion. This reinterpretation makes Europe a prime example of how technological and cultural change is linked with the expansion of a Y-chromosomal lineage, and the contrast of this pattern with that shown by maternally inherited mitochondrial DNA suggests a unique role for males in the transition.

## Abstract 2

Most present-day European men inherited their Y chromosomes from the farmers who spread from the Near East 10,000 years ago, rather than from the hunter-gatherers of the Paleolithic.

## Author Summary

Arguably the most important cultural transition in the history of modern humans was the development of farming, since it heralded the population growth that culminated in our current massive population size. The genetic diversity of modern populations retains the traces of such past events, and can therefore be studied to illuminate the demographic processes involved in past events. Much debate has focused on the origins of agriculture in Europe some 10,000 years ago, and in particular whether its westerly spread from the Near East was driven by farmers themselves migrating, or by the transmission of ideas and technologies to indigenous hunter-gatherers. This study examines the diversity of the paternally inherited Y chromosome, focusing on the commonest lineage in Europe. The distribution of this lineage, the diversity within it, and estimates of its age all suggest that it spread with farming from the Near East. Taken with evidence on the origins of other lineages, this indicates that most European Y chromosomes descend from Near Eastern farmers. In contrast, most maternal lineages descend from hunter-gatherers, suggesting a reproductive advantage for farming males over indigenous hunter-gatherer males during the cultural transition from hunting-gathering to farming.

## Introduction

**Figure 1:**
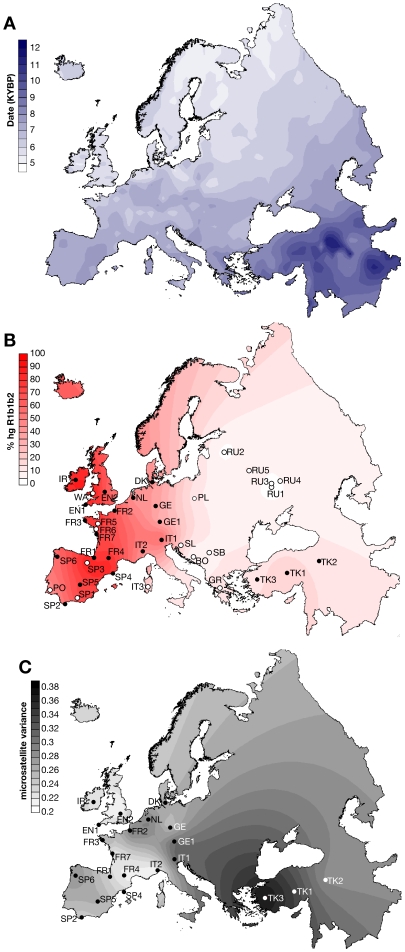
Maps showing dates of the spread of early farming in Europe, and the frequency and microsatellite variance of haplogroup R1b1b2. (A) Isochron map representing dates of early Neolithic sites in Europe, based on data of Pinhasi et al. (2005) [3]. KYBP, thousand years before present. (B) Geographical distribution of haplogroup frequency of hgR1b1b2, shown as an interpolated spatial frequency surface. Filled circles indicate populations for which microsatellite data and TMRCA estimates are available. Unfilled circles indicate populations included to illustrate R1b1b2 frequency only. Population codes are defined in Table 1. (C) Geographical distribution of mean microsatellite variance within hgR1b1b2, shown as an interpolated spatial frequency surface. Samples shown are those used for the calculation of variance only.

Events underlying the distribution of genetic diversity among modern European populations have been the subject of intense debate since the first genetic data became available [1]. Anatomically modern humans, originating in East Africa, colonized Europe from the Near East ∼40 thousand years ago (KYA), then during the last glacial maximum populations retreated into the peninsulas of Iberia, Italy, and the Balkans, followed by northward recolonization from these refugia ∼14 KYA. The most important cultural transition was the adoption of agriculture originating in the Fertile Crescent in the Near East at the start of the Neolithic, ∼10 KYA [2]. It spread rapidly westwards via Anatolia [3] (Figure 1A), reaching Ireland by 6 KYA, accompanied by the development of sedentary populations and demographic expansion. Debate has focused on whether this spread was due to the movement and expansion of Near-Eastern farmers (demic diffusion), or to the transmission of cultural innovation to existing populations (acculturation), who then themselves expanded.

The observation of southeast–northwest frequency clines for “classical” genetic markers [1],[4], autosomal DNA markers [5],[6], and Y-chromosomal markers [7],[8] (though not for mitochondrial DNA [mtDNA] [9]) has been used to support the demic diffusion model. No dates can be automatically attached to these clines, however, and some [1], detected by principal component analysis, may simply reflect isolation by distance [10]. The direction of movement underlying a cline can also be ambiguous: the high-frequency pole could indicate the area of preexisting substrate least affected by a migration originating far away, or the final destination of a wave of migration into thinly populated territory, where expansion and drift have had their greatest effects [11].

The origins of a frequency cline of a lineage can be illuminated by analysing the diversity within it. For Y-chromosomal lineages defined by binary markers (haplogroups), this can be done using multiple microsatellites. This approach has been applied to haplogroups E, J [12], and I [13] within Europe, but the major western European lineage has not yet been focused upon. The frequency of the major western European lineage, haplogroup (hg) R1b1b2, follows a cline from 12% in Eastern Turkey to 85% in Ireland (Figure 1B), and is currently carried by some 110 million European men. Previous studies of lineages approximately equivalent to hgR1b1b2 [7],[8] suggested that it has a Paleolithic origin, based simply on its high frequency in the west. Here, in contrast, we show that the geographical distribution of diversity within the haplogroup is best explained by its spread from a single source from the Near East via Anatolia during the Neolithic. Taken together with the evidence on the origins of many other European haplogroups, this indicates that the great majority of the Y chromosomes of Europeans have their origins in the Neolithic expansion.

## Results

**Figure 2:**
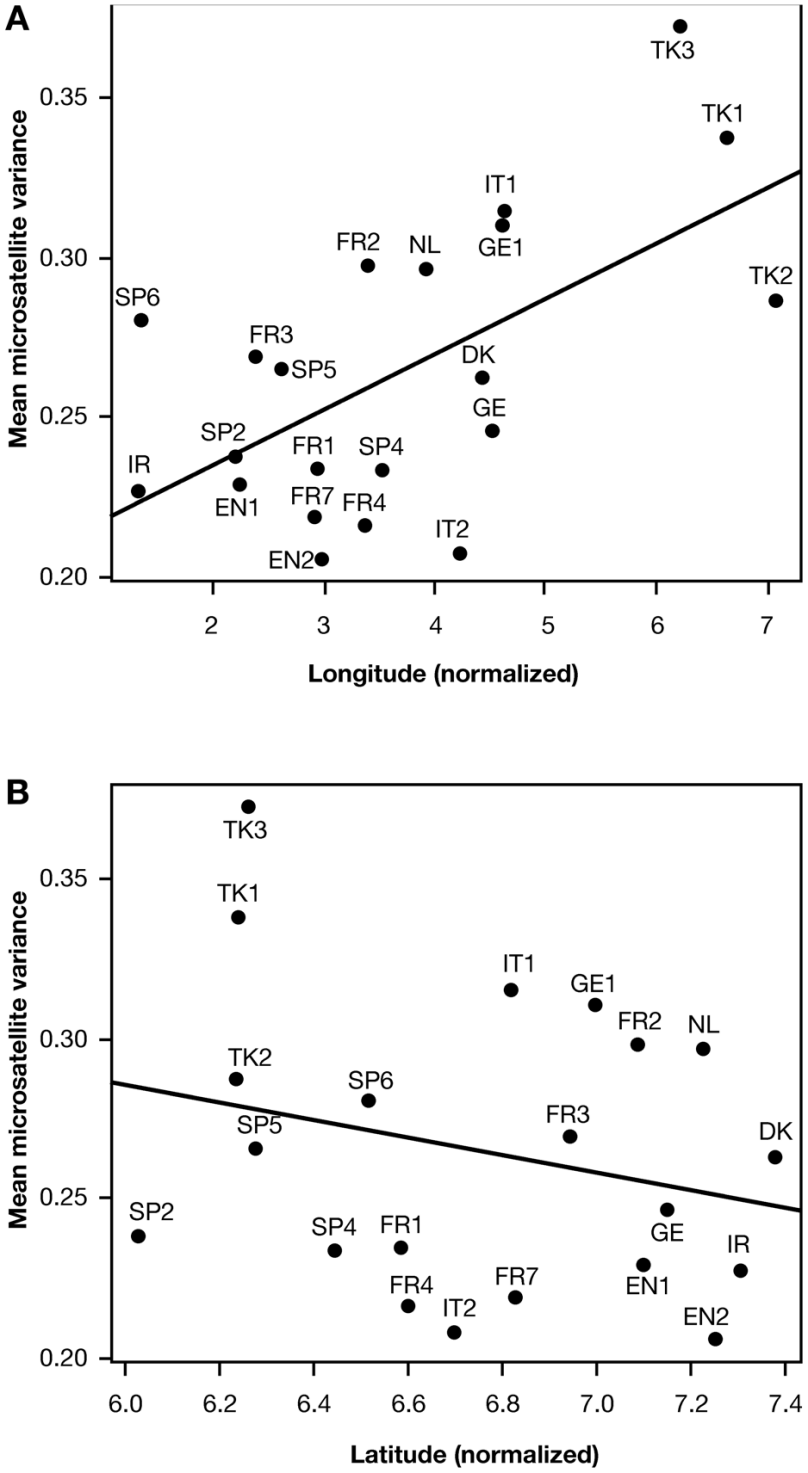
Relationship of diversity among 840 R1b1b2 chromosomes with (A) longitude and (B) latitude. Population codes are defined in Table 1.

To investigate the origins of hgR1b1b2, we assembled a dataset of 840 chromosomes from this haplogroup with associated nine-locus microsatellite haplotypes (Table 1; Table S1). The diversity of the lineage within each population (measured by mean microsatellite variance) should reflect its age: under a hypothesis of recolonization from southern refugia, we expect a gradient of diversity correlating with latitude, whereas Neolithic expansion from Anatolia predicts a correlation primarily with longitude. Figure 1C shows the geographical distribution of mean microsatellite variance, and Figure 2 shows that although there is no evidence for correlation with latitude (*R* ^2^ = 0.06; *p* = 0.268), the correlation with longitude is significant (*R* ^2^ = 0.358; *p* = 0.004), with greatest diversity in the east (strongly influenced by highly diverse samples within Turkey), thus providing support for the Neolithic colonization hypothesis.

**Table 1:** Frequency of haplogroup R1b1b2 in European populations, with geographical coordinates for sampled populations.

Country	Area of Sampling (Population)	Abbreviation	Included in All Analyses?	Longitude West	Latitude North	*N* a	% R1b1b2^b^	Source
Bosnia-Herzegovina	National	BO		17.650	43.850	256	3.9	[40]
Denmark	National	DK	Y	9.654	54.513	**56**	42.9	Present study
England	Cornwall	EN1	Y	−4.955	50.442	**64**	78.1	Present study
England	Leicestershire	EN2	Y	−1.130	52.637	**43**	62.0	Present study
France	Basques	FR1	Y	−1.305	43.384	**61**	75.4	Present study
France	Baie de Somme	FR2	Y	1.603	50.237	**43**	62.8	Present study
France	Finistère	FR3	Y	−4.264	48.233	**75**	76.0	Present study
France	Haute-Garonne	FR4	Y	1.443	43.604	**57**	78.9	Present study
France	Ile et Vilaine	FR5		−1.605	48.170	**82**	80.5	Present study
France	Loire-Atlantique	FR6		−1.741	47.348	**48**	77.1	Present study
France	Vendée	FR7	Y	−1.469	46.676	**50**	68.0	Present study
Germany	Bavaria	GE1	Y	11.319	48.985	**80**	32.3	Present study
Germany	National	GE	Y	10.451	51.165	1215	38.9	[41] c
Greece	National	GR		21.824	39.074	171	13.5	[42]
Italy	North-East (Ladin)	IT1	Y	11.552	46.528	**79**	60.8	Present study
Italy	North-West	IT2	Y	7.912	44.875	99	45.0	Present study
Ireland	National	IR	Y	−8.244	53.413	796	85.4	[43]
Italy	Sardinia	IT3		8.948	39.991	930	17.0	[44]
Netherlands	National	NL	Y	5.417	52.246	**84**	42.0	Present study
Poland	National	PL		19.145	51.919	913	11.6	[41] c
Portugal	South	PO		−8.176	37.750	**78**	46.2	Present study
Russia	Belgorod	RU1		36.480	50.780	143	2.8	[45]
Russia	Ostrov	RU2		28.320	57.350	75	2.7	[45]
Russia	Pristen	RU3		36.710	51.230	45	2.2	[45]
Russia	Repievka	RU4		38.650	51.080	96	5.2	[45]
Russia	Roslavl	RU5		32.870	53.950	107	11.2	[45]
Spain	Andalucia East	SP1		−3.209	37.513	**95**	72.0	Present study
Spain	Andalucia West	SP2	Y	−5.17	36.34	**72**	55.0	Present study
Spain	Basques	SP3		−2.430	42.580	**116**	87.1	Present study
Spain	Catalonia	SP4	Y	2.460	41.560	**80**	81.3	Present study
Spain	Castilla La Mancha	SP5	Y	−3.15	39.41	**63**	72.0	Present study
Serbia	National	SB		20.759	44.178	**100**	10.0	Present study
Spain	Galicia	SP6	Y	−8.150	42.510	**88**	58.0	Present study
Slovenia	National	SL		15.366	45.609	**70**	20.6	Present study
Turkey	Central	TK1	Y	34.036	38.942	152	19.1	[14]
Turkey	East	TK2	Y	40.110	38.921	208	12.0	[14]
Turkey	West	TK3	Y	28.570	39.243	163	13.5	[14]
Wales	National	WA		−3.793	52.170	**65**	92.3	Present study

a Figures in bold indicate samples typed for M269 in this study.

b The number of men currently carrying hgR1b1b2 chromosomes (see Introduction) was approximated from these proportions and population census sizes given at http://www.populationdata.net/europe.php.

c Chromosomes considered to belong to hgR1*(xR1a1).

Y, yes.

**Figure 3:**
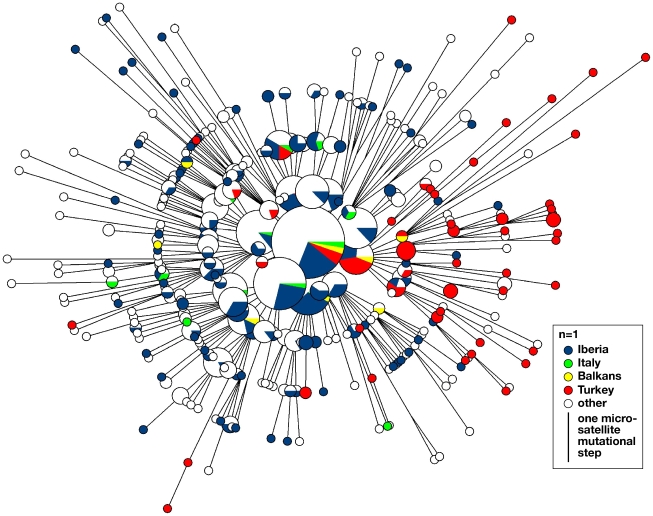
Reduced median network of microsatellite haplotypes within haplogroup R1b1b2. Molecular relationships between the nine-locus microsatellite haplotypes of 849 hgR1b1b2 chromosomes, including seven Serbian and two Greek haplotypes not included in the other analyses because population sample sizes were too small. Circles represent haplotypes, with area proportional to frequency and coloured according to population. Lines between circles represent microsatellite mutational steps.

The two hypotheses also make different predictions for the number of sources of diversity within hgR1b1b2: under the postglacial recolonization model, we expect multiple sources, whereas under the Neolithic expansion model, we expect only one. We can test this by examining the phylogenetic relationships among microsatellite haplotypes. A reduced median network of 859 haplotypes (Figure 3) shows a simple star-like structure indicative of expansion from one source: 74 haplotypes (8.6%) lie in its central node, and this node plus its single-step mutational neighbours together comprise 214 haplotypes (24.9%). Haplotypes belonging to populations from all three refugia are present in the core of the network. This pattern seems incompatible with recolonization from differentiated refugial populations, and in terms of the history of hgR1b1b2, the refugia possess no special status. The core of the network also contains haplotypes from Turkey (Anatolia), which is compatible with a subpopulation from this region acting as a source for the westwards-expanding lineage.

Does the time to the most recent common ancestor (TMRCA) of the hgR1b1b2 chromosomes support a Paleolithic origin? Mean estimates for individual populations vary (Table 2), but the oldest value is in Central Turkey (7,989 y [95% confidence interval (CI): 5,661–11,014]), and the youngest in Cornwall (5,460 y [3,764–7,777]). The mean estimate for the entire dataset is 6,512 y (95% CI: 4,577–9,063 years), with a growth rate of 1.95% (1.02%–3.30%). Thus, we see clear evidence of rapid expansion, which cannot have begun before the Neolithic period.

**Table 2:** Estimates of TMRCA for individual populations, arranged from west to east.

Population	TMRCA/y (mean [95% CI])
Ireland	5,533 (4,094–7,391)
Spain–Galicia	6,584 (4,923–8,684)
Spain–Andalucia West	6,208 (4,476–8,463)
England–Cornwall	5,460 (3,764–7,777)
France–Finistère	6,432 (4,786–8,571)
Spain–Castilla La Mancha	6,706 (4,772–9,261)
France–Vendée	6,787 (4,575–9,853)
France–Basques	5,797 (4,133–8,065)
England–Leicestershire	5,981 (4,051–8,439)
France–Haute–Garonne	5,925 (4,296–8,114)
France–Baie de Somme	7,384 (5,259–10,131)
Spain–Catalonia	5,800 (4,410–7,544)
Netherlands	6,952 (5,051–9,410)
Italy–North-West	5,944 (3,718–8,842)
Denmark	6,555 (4,391–9,386)
Germany–National	6,138 (4,627–7,997)
Germany–Bavaria	7,282 (5,059–10,139)
Italy–North-East (Ladin)	6,995 (4,635–10,396)
Turkey–West	7,304 (5,022–10,359)
Turkey–Central	7,989 (5,661–11,014)
Turkey–East	7,000 (4,423–10,490)

**Figure 4:**
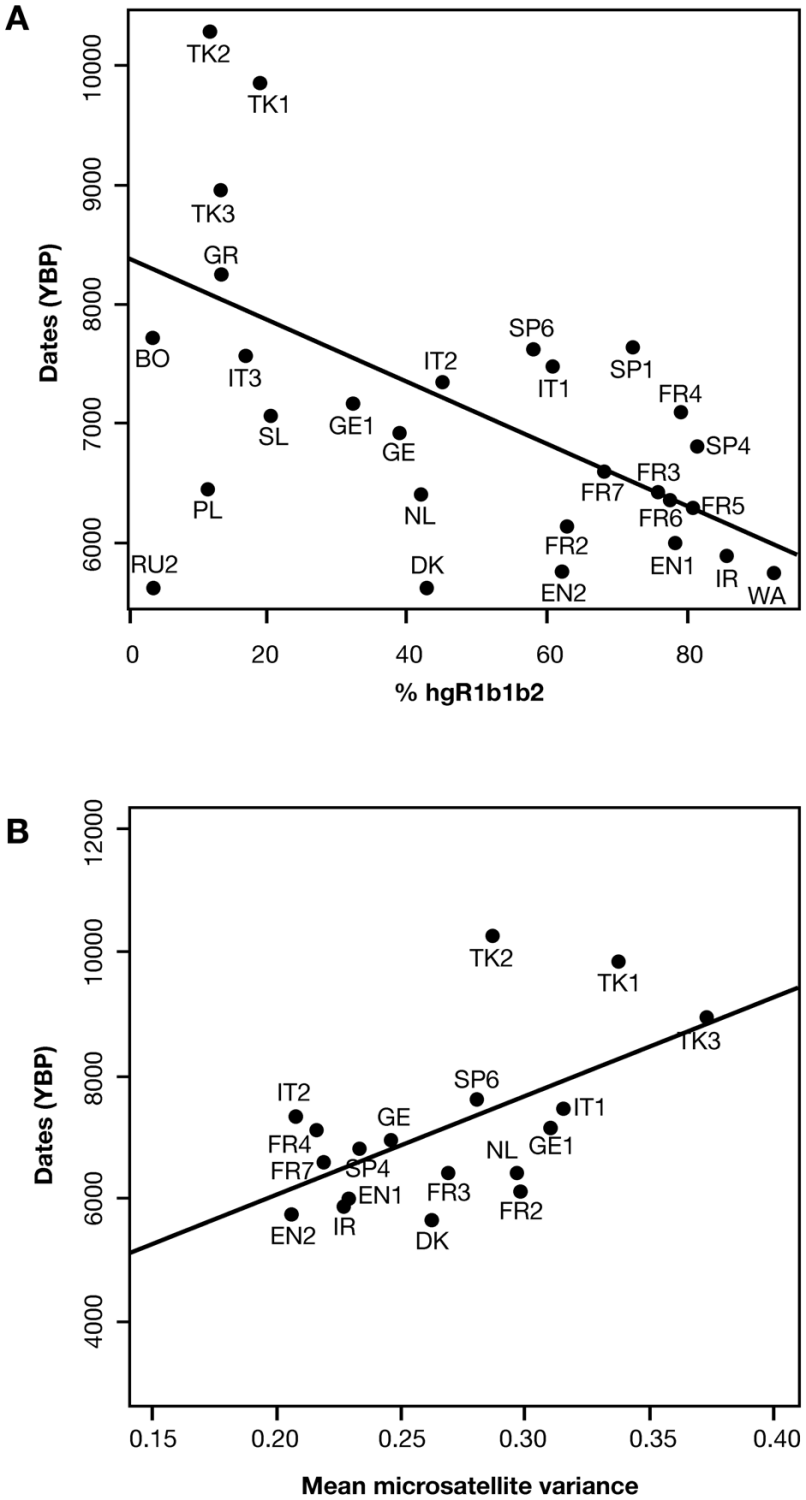
Correlation of dates of Neolithic sites with hgR1b1b2 (A) frequency and (B) variance. Population codes are defined in Table 1. YBP, years before present.

The similarity between the isochron map of Neolithic sites (Figure 1A; [3]) and those of hgR1b1b2 frequency (Figure 1B) and diversity (Figure 1C) is striking. Further support for the association of the expansion of hgR1b1b2 with that of farming comes from a statistical comparison of the variables. The frequency of hgR1b1b2 at different points in Europe is significantly negatively correlated (*R* ^2^ = 0.390; *p* = 0.0005) with the dates of local Neolithic sites (Figure 4A). For the local variance of the microsatellite haplotypes within hgR1b1b2, the correlation with Neolithic dates is significantly positive (*R* ^2^ = 0.331; *p* = 0.0124; Figure 4B).

## Discussion

Previous observations of the east–west clinal distribution of the common Western European hgR1b1b2 (or its equivalent) [7],[8] considered it to be part of a Paleolithic substrate into which farmers from the Near East had diffused. Later analyses have also considered variance, and have conformed to the Paleolithic explanation [14],[15]. Here, we concur that the cline results from demic diffusion, but our evidence supports a different interpretation: that R1b1b2 was carried as a rapidly expanding lineage from the Near East via Anatolia to the western fringe of Europe during the Neolithic. Such mutations arising at the front of a wave of expansion have a high probability of surviving and being propagated, and can reach high frequencies far from their source [11]. Successive founder effects at the edge of the expansion wave can lead to a reduction in microsatellite diversity, even as the lineage increases in frequency.

The innovations in the Near East also spread along the southern shore of the Mediterranean, reflected in the expansion of hgE1b1b1b (E-M81) [16], which increases in frequency and reduces in diversity from east to west. In sub-Saharan Africa, hgE1b1a (E-M2) underwent a massive expansion associated with the Bantu expansion [17],[18]. In India, the spread of agriculture has been associated with the introduction of several Y lineages [19], and in Japan, lineages within hgO spread with the Yayoi migration [20], which brought wet rice agriculture to the archipelago. On a more recent timescale, the expansion of the Han culture in China has been linked to demic diffusion [21]. In this context, the apparently low contribution of incoming Y chromosomes to the European Neolithic, despite its antiquity and impact, has appeared anomalous. Our interpretation of the history of hgR1b1b2 now makes Europe a prime example of how expansion of a Y-chromosomal lineage tends to accompany technological and cultural change.

Other lineages also show evidence of European Neolithic expansion, hgE1b1b1 (E-M35) and hgJ, in particular [12]. Indeed, hgI is the only major lineage for which a Paleolithic origin is generally accepted, but it comprises only 18% of European Y chromosomes [13]. The Basques contain only 8%–20% of this lineage, but 75%–87% hgR1b1b2 (Table S1); our findings therefore challenge their traditional “Mesolithic relict” status, and in particular, their use as a proxy for a Paleolithic parental population in admixture modelling of European Y-chromosomal prehistory [22].

Is the predominance of Neolithic-expansion lineages among Y chromosomes reflected in other parts of the genome? Mitochondrial DNA diversity certainly presents a different picture: no east–west cline is discernible, most lineages have a Paleolithic TMRCA [23], and hgH [24] and hgV [25] show signatures of postglacial expansion from the Iberian peninsula. Demic diffusion involves both females and males, but the disparity between mtDNA and Y-chromosomal patterns could arise from an increased and transmitted reproductive success for male farmers compared to indigenous hunter-gatherers, without a corresponding difference between females from the two groups. This would lead to the expansion of incoming Y lineages—as suggested by the high growth rate observed for hgR1b1b2. Similar conclusions have been reached for the Bantu expansion (in which the current Bantu-speaking populations carry many mtDNA lineages originating from hunter-gatherers [26]), the introduction of agriculture to India [19] and the Han expansion [21].

Some studies have found evidence of east–west clines for autosomal loci [6],[27]. By contrast, recent genome-wide SNP typing surveys [28]–[30] find a basic south–north division or gradient, including greater diversity in the south, but they provide no indication of the time-depth of the underlying events, which could in principle involve contributions from the original colonization, postglacial Paleolithic recolonization, Neolithic expansion, and later contact between Africa and southern Europe [31].

The distinction between the geographical patterns of variation of the Y chromosome and those of mtDNA suggest sex-specific factors in patterning European diversity, but the rest of the genome has yet to reveal definitive information. Detailed studies of X-chromosomal and autosomal haplotypes promise to further illuminate the roles of males and females in prehistory.

## Materials and Methods

### Ethics Statement

Males were recruited with informed consent, following ethical approval by the Leicestershire Research Ethics Committee and the ethics committees of the Universities of Ferrara, Pavia, and Exeter and Plymouth.

### DNA Samples and Haplotyping

A total of 2,574 DNA samples from European males, assigned to populations based on two generations of residence, were typed for the SNP M269 [17], defining hgR1b1b2. Following PCR amplification using the primers 5′-CTAAAGATCAGAGTATCTCCCTTTG-3′ and 5′-ATTTCTAGGAGTTCACTGTATTAC-3′, the T to C transition was analysed by digestion with BstNI, which cleaves M269-C-allele chromosomes only. Samples from the Iberian peninsula were typed using the SNaPshot (ABI) procedure [31]. Haplotype data were obtained for up to 20 Y-specific microsatellites [32],[33]. Data from the Ysearch database (http://www.ysearch.org) for Germany (GE) and Ireland (IR) were added, together with published data for Turkey, subdivided into East, West, and Central subpopulations based on published sampling information [14]. To avoid a bias from very large samples of hgR1b1b2 (GE and IR), these were randomly subsampled to give sample sizes of 75. This allowed a comparison of nine-locus haplotypes (DYS19, DYS388, DYS389I, DYS389II, DYS390, DYS391, DYS392, DYS393, and DYS439) for 849 hgR1b1b2 chromosomes, subdivided into 23 populations. Greek and Serbian samples were too small for population-based analyses, but were included in Network analysis.

### Analysis

Neolithic dates, frequencies of hgR1b1b2, and local microsatellite variances were displayed using Surfer 8.02 (Golden Software) by the gridding method. Latitudes and longitudes were based on sampling centres.

Intrahaplogroup diversity was assessed for populations with hgR1b1b2 sample size ≥15 as the mean of the individual microsatellite variances [34], as has been done elsewhere (e.g., [35]); this measure is highly correlated (*R* ^2^ = 0.871; *p* = 6.72×10^−10^) with a more conventional measure, average squared distance (ASD) [36]. Regression analyses were carried out in the R statistical package [37] to compare these two measures, and also to compare mean of variance with latitude and longitude.

A reduced median network [38] of microsatellite haplotypes was constructed using Network 4.5 and Network Publisher, using weighting based on the inverse of the microsatellite variances.

TMRCA and population growth rates were estimated using BATWING [39], under a model of exponential population growth and splitting. Whereas standard use of BATWING assumes a random sample from a population, we validated its use to analyse single haplogroups. Justification of this, together with other details, is given in Text S1.

To assess the correlation between the dates of Neolithic sites and the local hgR1b1b2 frequency and variance, we considered 765 sites and their associated calibrated radiocarbon dates [3]. We identified sites lying within a buffer-zone of 150-km radius around each location for which we had frequency or variance data (Figure 1B and 1C). When more than one site was identified in a given buffer-zone, we considered the mean of the dates. Regression analyses were carried out as described above.

## Supporting Information

Table S1 **Haplotype data.** Population abbreviations are as in Table 1; for each microsatellite (DYS19–DYS439), repeat unit numbers are given.
(0.14 MB PDF)

Text S1 **Details of application of BATWING.**
(0.14 MB DOC)

## Abbreviations

hg: haplogroup
KYA: thousand years ago
mtDNA: mitochondrial DNA
TMRCA: time to the most recent common ancestor
