# Supplementary material for: A Predominantly Neolithic Origin for European Paternal Lineages
Source: PLoS Biol. 2010 Jan 19;8(1):e1000285. doi: 10.1371/journal.pbio.1000285 (PMC2799514; doi:10.1371/journal.pbio.1000285)
Supplement: Table S1 — Haplotype data. Population abbreviations are as in Table 1; for each microsatellite (DYS19–DYS439), repeat unit numbers are given. (0.14 MB PDF) [file pbio.1000285.s001.pdf]

Population	All analyses?	DYS19	DYS388	DYS389I	DYS389b	DYS390	DYS391	DYS392	DYS393	DYS439
DK	y	14	12	13	15	23	11	14	13	19
DK	y	14	12	13	16	24	11	13	13	18
DK	y	14	12	14	16	24	10	13	13	20
DK	y	14	12	13	16	24	12	13	13	18
DK	y	14	12	13	16	24	11	13	12	19
DK	y	14	12	12	16	23	11	13	13	19
DK	y	14	12	13	16	24	10	13	14	19
DK	y	14	12	13	16	25	11	13	13	19
DK	y	14	12	13	16	23	10	13	13	19
DK	y	14	12	13	17	25	11	13	13	19
DK	y	14	12	14	15	23	11	13	13	18
DK	y	14	12	12	16	23	11	13	13	19
DK	y	14	12	13	16	24	11	13	13	20
DK	y	14	12	13	17	23	10	13	13	19
DK	y	14	12	13	16	23	11	13	13	19
DK	y	14	12	13	16	23	11	13	13	18
DK	y	14	12	13	15	23	12	13	13	18
DK	y	14	12	14	16	24	11	13	13	19
DK	y	15	12	13	15	25	10	13	13	20
GE1	y	14	13	12	16	24	11	13	13	18
GE1	y	14	12	13	16	23	11	13	13	19
GE1	y	14	12	13	17	24	10	13	12	19
GE1	y	14	12	13	16	24	11	14	13	19
GE1	y	14	12	13	16	23	11	13	13	19
GE1	y	14	12	13	16	24	10	14	14	19
GE1	y	14	12	13	16	24	11	13	14	20
GE1	y	15	12	13	16	23	11	13	13	19
GE1	y	14	12	12	17	23	11	13	13	18
GE1	y	15	12	13	16	25	11	13	12	19
GE1	y	15	12	14	16	24	11	13	12	19
GE1	y	14	12	13	16	23	10	13	13	19
GE1	y	14	12	12	19	24	11	13	12	19
GE1	y	14	12	13	17	24	11	13	12	19
GE1	y	14	12	13	16	24	10	13	13	18
GE1	y	15	12	13	16	24	10	13	13	19
GE1	y	15	12	12	15	24	10	13	13	18
GE1	y	14	12	13	16	24	11	13	13	19
GE1	y	14	12	13	16	23	11	13	12	21
NL	y	14	12	14	16	24	10	13	13	19
NL	y	14	12	13	15	23	10	13	13	18
NL	y	14	12	13	17	24	10	15	13	20
NL	y	14	12	13	15	23	11	13	13	18
NL	y	14	12	13	16	24	11	13	13	19
NL	y	14	12	14	16	24	11	13	13	19
NL	y	13	12	13	15	23	12	13	13	18
NL	y	14	12	14	16	23	11	13	13	18
NL	y	14	12	13	17	24	11	13	13	20
NL	y	14	12	13	16	24	11	13	14	19
NL	y	14	12	13	15	24	10	13	13	20
NL	y	14	12	13	17	26	10	13	13	20
NL	y	14	12	13	16	23	10	13	13	18
NL	y	14	12	12	16	24	10	13	13	19
NL	y	14	12	13	16	24	10	13	13	19
NL	y	14	12	13	16	24	11	13	13	19
NL	y	14	12	13	16	23	12	13	13	19
NL	y	15	12	13	15	24	10	13	14	18
NL	y	15	12	13	16	24	11	13	13	18
NL	y	14	12	13	16	24	11	13	13	19
NL	y	14	12	14	15	23	11	13	13	19
NL	y	14	12	14	16	23	10	13	13	20
NL	y	14	12	13	16	25	10	14	12	19
NL	y	14	12	13	16	23	11	13	13	19
NL	y	14	12	13	15	23	12	13	13	20
NL	y	14	12	12	16	24	11	13	13	19
NL	y	14	12	13	16	23	11	13	12	18
NL	y	14	12	13	17	25	10	13	12	18
NL	y	14	12	13	16	24	11	13	13	19
NL	y	14	12	13	16	24	11	13	13	19
NL	y	14	12	13	15	23	11	13	13	18
NL	y	14	12	13	16	25	10	13	13	19
NL	y	14	12	13	16	25	11	13	13	19
NL	y	14	12	13	14	24	11	13	13	19
IT2	y	15	12	13	16	23	10	13	13	20
IT2	y	14	12	13	17	25	11	13	13	19
IT2	y	14	11	13	16	24	10	15	13	19
IT2	y	14	12	13	16	23	11	13	14	19
IT2	y	14	12	13	16	24	10	13	13	19
IT2	y	14	12	12	16	24	10	13	13	20
IT2	y	14	12	14	16	24	11	13	13	18
IT2	y	14	12	13	16	23	11	13	13	20
IT2	y	14	12	13	16	24	11	13	13	19
IT2	y	14	13	13	16	24	11	13	13	19
IT2	y	15	12	13	16	23	10	13	13	19
IT2	y	14	12	13	16	24	11	13	13	20
IT2	y	14	12	13	16	23	11	13	13	19
IT2	y	14	12	13	16	24	11	13	13	19
IT2	y	14	12	13	17	24	10	13	13	19
IT1	y	14	12	13	16	23	10	13	13	18
IT1	y	14	12	13	16	25	11	13	14	19
IT1	y	14	12	13	16	23	10	13	13	19
IT1	y	14	12	13	16	24	11	13	13	19
IT1	y	13	12	13	16	23	10	13	13	20
IT1	y	14	12	13	17	25	11	13	14	19
IT1	y	14	12	13	16	24	11	13	13	20
IT1	y	14	12	13	16	23	9	13	13	18
IT1	y	14	12	13	16	25	10	13	13	19
IT1	y	14	12	13	16	23	10	13	13	18
IT1	y	14	12	13	16	25	10	13	13	19

Population	All analyses?	DYS19	DYS388	DYS389I	DYS389b	DYS390	DYS391	DYS392	DYS393	DYS439
IT1	y	14	12	13	16	23	11	13	13	19
IT1	y	14	12	13	16	23	10	13	13	18
IT1	y	14	12	13	16	23	10	13	13	18
IT1	y	14	12	13	16	24	10	13	13	19
IT1	y	14	12	13	16	25	10	13	13	19
IT1	y	14	12	13	16	23	11	13	13	18
IT1	y	14	12	13	16	25	11	13	13	19
IT1	y	14	12	13	17	21	12	13	13	19
IT1	y	14	12	13	17	23	10	13	13	18
IT1	y	14	12	13	16	23	10	13	13	18
IT1	y	14	12	13	16	25	12	13	13	20
IT1	y	14	12	14	16	23	10	13	13	18
IT1	y	14	12	12	17	24	11	13	13	19
IT1	y	14	12	12	16	23	11	13	13	18
IT1	y	14	12	13	17	23	12	13	13	19
IT1	y	14	12	13	16	23	10	13	13	18
IT1	y	14	12	13	16	25	9	13	12	19
IT1	y	14	12	13	16	23	10	13	13	18
IT1	y	14	12	13	16	24	11	13	13	19
IT1	y	14	12	13	16	25	11	13	12	19
IT1	y	14	12	13	16	24	11	13	13	19
IT1	y	14	12	13	16	23	11	13	13	19
IT1	y	14	15	13	16	24	11	13	12	20
IT1	y	14	13	13	17	24	10	13	13	19
IT1	y	14	15	13	16	24	11	13	12	21
IT1	y	14	12	13	16	23	11	13	13	19
IT1	y	14	12	13	16	25	10	13	14	19
IT1	y	14	12	14	17	24	10	13	13	20
IT1	y	14	12	13	17	24	10	13	13	19
IT1	y	14	12	13	17	24	10	13	13	19
IT1	y	14	12	13	17	24	10	13	13	19
IT1	y	14	12	13	17	24	10	13	13	19
IT1	y	14	12	13	16	24	11	13	13	19
IT1	y	14	12	14	16	24	11	13	13	20
IT1	y	14	12	13	16	24	11	13	13	19
IT1	y	14	12	13	16	24	11	14	13	19
EN1	y	14	12	13	16	24	10	13	13	19
EN1	y	14	12	13	16	23	11	13	13	19
EN1	y	14	12	13	16	23	11	13	13	19
EN1	y	14	12	12	16	23	11	13	14	20
EN1	y	15	12	13	16	24	11	13	13	20
EN1	y	14	12	13	16	24	11	13	13	19
EN1	y	14	12	13	16	24	10	13	13	19
EN1	y	14	12	12	16	24	11	13	13	19
EN1	y	14	12	14	16	24	11	13	13	19
EN1	y	14	12	13	16	23	11	13	13	20
EN1	y	14	12	14	16	25	11	13	13	19
EN1	y	14	12	13	16	24	11	13	13	18
EN1	y	14	12	14	16	24	11	13	13	19
EN1	y	14	12	13	16	24	11	13	14	19
EN1	y	14	12	15	16	23	11	13	13	19
EN1	y	14	12	13	17	24	11	13	13	20
EN1	y	14	12	13	16	24	11	13	13	18
EN1	y	14	12	13	16	23	11	13	13	20
EN1	y	14	12	14	16	24	11	13	13	19
EN1	y	14	12	13	17	24	11	13	13	19
EN1	y	14	12	13	16	23	11	13	13	19
EN1	y	14	12	13	16	24	11	14	13	19
EN1	y	14	12	13	15	24	10	13	13	19
EN1	y	14	12	13	17	24	11	13	13	19
EN1	y	14	12	13	16	23	11	13	13	19
EN1	y	14	12	12	16	24	11	13	13	18
EN1	y	14	12	13	17	24	11	13	13	20
EN1	y	14	12	13	16	24	11	13	13	20
EN1	y	14	12	14	16	26	11	13	12	19
EN1	y	14	12	13	16	24	11	13	13	19
EN1	y	14	12	12	15	24	11	13	13	19
EN1	y	14	12	13	16	23	11	13	13	19
EN1	y	14	12	13	16	23	11	13	13	18
EN1	y	14	12	14	16	23	11	13	13	19
EN1	y	14	12	14	16	23	11	13	13	19
EN1	y	14	12	12	17	24	11	13	13	19
EN1	y	14	12	13	16	24	11	13	13	19
EN1	y	14	12	13	16	24	11	13	13	19
EN1	y	14	12	13	16	24	11	13	13	19
EN1	y	14	12	13	17	24	11	13	13	19
EN1	y	14	12	13	16	24	10	13	13	21
EN1	y	14	12	13	17	23	11	13	12	20
EN1	y	14	12	14	16	26	11	13	12	19
EN1	y	14	11	13	16	23	11	13	13	18
EN1	y	14	12	14	16	23	10	13	13	19
EN1	y	14	12	13	16	24	11	13	14	19
EN1	y	14	12	13	16	24	10	13	13	20
EN1	y	14	12	14	16	26	11	13	12	19
EN1	y	14	12	13	18	23	11	13	13	19
FR7	y	14	12	13	17	24	10	13	13	19
FR7	y	14	12	13	16	24	10	13	14	19
FR7	y	14	12	14	15	24	12	13	13	20
FR7	y	13	12	13	16	23	11	13	13	18
FR7	y	14	12	13	16	24	11	13	13	19
FR7	y	14	12	13	16	24	11	13	13	19
FR7	y	14	12	13	17	24	11	13	13	18
FR7	y	14	12	13	16	24	11	13	13	20
FR7	y	15	13	13	16	25	12	12	13	19
FR7	y	15	13	13	17	24	10	13	13	19
FR7	y	14	12	13	16	24	11	13	13	20
FR7	y	14	12	13	16	24	11	13	13	19
FR7	y	14	12	13	16	24	11	13	13	19

Population	All analyses?	DYS19	DYS388	DYS389I	DYS389b	DYS390	DYS391	DYS392	DYS393	DYS439
FR7	y	14	12	13	16	24	10	13	13	19
FR7	y	15	12	13	17	23	11	13	13	20
FR7	y	15	12	13	17	23	11	13	13	20
FR7	y	14	12	13	17	24	10	13	13	19
FR7	y	14	12	13	16	23	10	13	13	19
FR7	y	14	12	13	18	25	11	13	13	19
FR7	y	14	12	13	17	24	11	13	13	19
FR7	y	14	12	13	17	24	11	13	13	19
FR7	y	13	12	13	17	24	11	13	14	19
FR7	y	14	12	14	16	24	11	13	13	18
FR7	y	14	12	13	16	24	11	12	14	18
FR7	y	14	12	13	17	24	11	13	14	18
FR7	y	14	12	13	16	24	11	13	13	19
FR7	y	14	12	13	18	25	11	13	13	19
FR7	y	14	12	14	16	25	10	13	13	19
FR7	y	14	12	13	16	24	11	13	13	20
FR7	y	14	12	13	17	24	10	13	13	19
FR7	y	14	12	13	16	24	10	13	13	18
FR7	y	15	12	13	16	24	11	13	13	18
FR7	y	14	12	13	16	24	10	13	13	18
FR3	y	14	12	13	17	24	10	13	13	19
FR3	y	14	12	14	16	24	10	14	13	19
FR3	y	14	12	13	17	24	11	13	12	19
FR3	y	14	12	13	16	24	10	14	13	21
FR3	y	14	12	13	16	24	11	13	13	18
FR3	y	15	12	13	16	25	10	13	14	19
FR3	y	16	12	13	16	24	10	13	13	19
FR3	y	14	12	13	16	24	10	13	13	19
FR3	y	14	12	13	16	24	10	13	13	19
FR3	y	14	12	13	16	24	11	14	13	19
FR3	y	14	12	13	16	24	10	13	13	19
FR3	y	14	12	14	16	24	10	13	13	20
FR3	y	14	12	12	16	24	11	13	13	18
FR3	y	14	12	15	17	23	10	13	14	19
FR3	y	14	12	13	16	22	11	13	13	18
FR3	y	14	12	13	16	24	11	13	12	19
FR3	y	14	12	13	15	24	11	13	13	19
FR3	y	14	12	13	16	24	10	13	13	19
FR3	y	14	12	13	17	24	11	13	12	19
FR3	y	14	12	13	16	25	11	13	12	19
FR3	y	15	12	13	16	24	10	13	13	19
FR3	y	14	12	13	16	21	11	13	13	18
FR3	y	14	13	14	16	24	11	13	13	20
FR3	y	14	12	14	16	25	11	13	13	19
FR3	y	14	12	13	17	24	10	13	13	18
FR3	y	14	12	13	17	25	12	13	13	19
FR3	y	14	12	13	16	23	11	15	13	19
FR3	y	14	12	13	16	24	11	14	13	19
FR3	y	14	12	13	16	24	11	13	13	19
FR3	y	14	12	13	16	24	11	13	13	19
FR3	y	14	12	12	17	24	11	13	13	19
FR3	y	15	12	12	16	24	10	13	13	19
FR3	y	14	13	14	16	24	11	13	13	20
FR3	y	15	12	13	17	24	10	13	13	19
FR3	y	14	12	13	16	24	10	13	13	20
FR3	y	14	12	13	17	24	11	13	13	19
FR3	y	15	12	13	16	24	11	13	13	18
FR3	y	14	12	14	17	24	10	13	13	19
FR3	y	14	12	13	16	23	11	13	13	20
FR3	y	15	12	12	16	23	10	13	14	19
FR3	y	14	12	14	17	24	10	13	13	19
FR3	y	14	12	13	17	24	10	13	13	19
FR3	y	15	12	12	16	24	11	13	13	19
FR3	y	15	12	13	16	24	10	13	13	19
FR3	y	14	12	13	16	23	11	13	13	18
FR3	y	14	12	13	16	23	11	13	13	19
FR3	y	14	12	13	16	24	11	11	13	20
FR3	y	15	12	12	16	24	10	13	13	19
FR3	y	14	12	13	16	25	11	13	13	19
FR3	y	14	12	13	16	24	10	13	13	18
FR3	y	14	12	13	16	24	10	13	13	19
FR3	y	14	12	14	16	24	11	13	13	19
FR3	y	14	12	13	17	24	11	13	13	19
FR3	y	15	12	13	16	25	10	13	14	19
FR3	y	14	12	13	16	23	11	14	13	20
FR3	y	14	12	13	18	24	10	13	13	20
FR3	y	16	12	13	17	24	12	13	14	18
FR2	y	14	12	13	16	23	11	13	13	19
FR2	y	14	12	14	16	23	11	11	13	19
FR2	y	14	12	13	16	25	11	13	13	19
FR2	y	14	12	13	16	24	10	13	14	19
FR2	y	15	12	13	16	23	11	13	13	19
FR2	y	14	12	12	16	23	10	13	13	19
FR2	y	14	12	13	16	24	11	13	13	19
FR2	y	14	12	13	16	25	10	14	13	20
FR2	y	14	13	13	16	24	11	13	13	19
FR2	y	15	12	13	18	24	11	12	12	19
FR2	y	14	12	13	16	24	11	13	13	20
FR2	y	14	12	13	16	24	11	13	13	19
FR2	y	14	12	13	16	23	11	13	13	20
FR2	y	14	12	14	17	24	10	13	12	19
FR2	y	14	12	13	16	25	11	13	13	19
FR2	y	14	12	12	15	24	10	13	13	20
FR2	y	14	12	13	16	24	10	13	13	18
FR2	y	14	12	13	16	23	11	13	13	18
FR2	y	14	12	13	16	23	11	14	13	19
FR2	y	14	12	13	16	24	10	13	13	19
FR2	y	14	12	13	16	24	11	13	13	18

Population	All analyses?	DYS19	DYS388	DYS389I	DYS389b	DYS390	DYS391	DYS392	DYS393	DYS439
FR2	y	13	12	13	16	24	10	13	13	19
FR2	y	15	12	12	18	24	10	13	13	19
FR2	y	15	12	13	16	24	11	13	14	20
FR2	y	14	12	11	16	25	12	13	14	20
FR2	y	14	12	13	16	23	12	13	13	19
FR2	y	13	12	14	17	24	11	13	13	19
EN2	y	14	12	13	16	24	11	13	13	19
EN2	y	14	12	11	16	24	10	13	13	19
EN2	y	14	12	13	16	23	11	13	13	20
EN2	y	15	12	13	16	24	11	13	13	18
EN2	y	14	12	13	16	23	11	13	13	19
EN2	y	14	12	13	16	24	11	13	13	19
EN2	y	14	12	14	16	23	10	13	13	19
EN2	y	14	12	14	16	24	11	13	13	19
EN2	y	14	12	13	17	23	11	13	13	20
EN2	y	15	12	13	16	23	12	13	13	19
EN2	y	15	13	14	16	23	11	13	13	19
EN2	y	14	12	13	16	23	11	13	13	18
EN2	y	14	12	13	16	24	11	13	13	19
EN2	y	14	12	13	16	24	11	13	13	19
EN2	y	14	12	13	16	24	10	13	13	19
EN2	y	14	12	13	16	23	11	13	13	18
EN2	y	14	12	12	16	25	12	13	13	19
EN2	y	14	12	13	16	24	10	13	13	18
EN2	y	13	12	13	16	24	11	13	14	20
EN2	y	14	12	13	16	24	10	13	13	19
EN2	y	14	12	13	17	23	11	13	13	18
EN2	y	15	12	13	16	23	10	13	13	20
EN2	y	14	12	13	16	25	11	14	13	19
EN2	y	14	12	13	16	23	11	13	13	19
EN2	y	14	12	13	16	24	10	13	13	19
FR1	y	14	12	13	16	24	10	13	13	19
FR1	y	14	12	13	16	24	10	13	13	18
FR1	y	14	12	13	16	24	10	13	13	19
FR1	y	13	12	14	17	24	11	13	13	19
FR1	y	14	12	13	17	24	11	13	13	18
FR1	y	14	12	13	16	25	10	13	13	19
FR1	y	14	12	14	17	24	10	13	13	19
FR1	y	14	12	13	16	23	10	13	13	18
FR1	y	14	12	13	18	24	11	13	13	20
FR1	y	14	12	13	16	24	10	13	13	19
FR1	y	14	12	13	16	23	11	13	13	19
FR1	y	14	12	13	16	24	10	13	13	19
FR1	y	14	12	14	16	24	8	13	13	19
FR1	y	14	12	13	18	24	11	13	13	18
FR1	y	13	14	13	16	24	10	13	13	19
FR1	y	13	14	13	16	24	10	13	13	19
FR1	y	14	12	13	16	24	11	13	13	21
FR1	y	15	12	14	16	24	11	13	13	19
FR1	y	14	12	13	16	24	10	13	13	18
FR1	y	15	12	14	16	24	10	13	13	19
FR1	y	14	12	14	16	24	11	13	13	19
FR1	y	14	12	13	16	24	11	13	13	19
FR1	y	14	12	13	16	24	10	13	13	19
FR1	y	14	12	14	16	24	11	13	13	21
FR1	y	14	12	13	16	24	10	13	13	19
FR1	y	14	12	13	16	24	10	13	13	20
FR1	y	14	12	13	17	24	11	13	13	20
FR1	y	14	12	14	16	23	11	13	13	19
FR1	y	14	12	13	16	23	10	13	13	19
FR1	y	14	12	14	16	23	11	12	13	18
FR1	y	14	12	14	16	23	10	13	13	19
FR1	y	14	12	14	15	24	11	13	13	20
FR1	y	14	12	13	17	24	11	13	13	19
FR1	y	14	12	14	16	24	10	13	13	19
FR1	y	14	12	14	17	24	12	13	13	19
FR1	y	14	12	14	17	24	12	13	13	19
FR1	y	14	12	13	16	24	11	13	13	19
FR1	y	14	12	14	17	24	11	13	13	19
FR1	y	14	12	13	16	24	11	13	13	19
FR1	y	14	12	13	16	24	10	13	13	20
FR1	y	13	12	12	16	24	11	13	13	19
FR1	y	13	12	13	16	24	10	13	13	19
FR1	y	15	12	13	16	24	10	13	13	19
FR1	y	14	12	14	16	24	11	13	13	20
FR1	y	14	12	13	16	24	10	13	14	19
FR1	y	14	12	13	16	24	10	13	13	20
IR	y	14	12	14	17	24	11	13	13	19
IR	y	14	12	13	16	24	11	13	13	20
IR	y	14	12	14	16	24	11	13	13	19
IR	y	15	12	13	16	25	11	13	13	18
IR	y	14	12	13	17	24	11	13	13	18
IR	y	14	12	13	16	24	11	13	13	19
IR	y	14	12	14	17	24	10	13	13	19
IR	y	14	12	13	16	24	11	13	13	18
IR	y	14	12	13	16	24	12	13	13	20
IR	y	14	12	13	16	24	10	13	12	19
IR	y	14	12	13	16	24	11	13	13	18
IR	y	14	12	14	16	24	11	13	13	18
IR	y	14	12	14	16	24	11	13	13	19
IR	y	14	12	13	16	24	9	13	13	18
IR	y	14	12	13	16	25	11	13	13	19
IR	y	14	12	14	17	24	10	13	13	18
IR	y	14	12	13	16	24	11	13	13	20
IR	y	14	12	14	16	24	10	13	13	19
IR	y	14	12	13	17	24	10	13	13	19
IR	y	14	12	13	16	23	11	13	14	19
IR	y	14	12	13	16	25	11	13	13	19

Population	All analyses?	DYS19	DYS388	DYS389I	DYS389b	DYS390	DYS391	DYS392	DYS393	DYS439
IR	y	14	12	13	15	23	10	13	13	18
IR	y	14	12	13	16	24	11	13	13	20
IR	y	14	12	14	16	24	11	13	13	19
IR	y	14	12	14	17	24	11	13	13	19
IR	y	15	12	13	15	23	12	13	13	18
IR	y	14	12	14	16	24	11	13	13	19
IR	y	14	12	13	16	23	11	13	13	20
IR	y	14	12	13	16	24	10	14	13	19
IR	y	14	12	13	15	23	10	13	13	19
IR	y	14	12	13	16	23	11	13	13	19
IR	y	14	12	13	16	24	10	13	13	19
IR	y	14	12	13	17	24	10	13	13	19
IR	y	14	12	13	16	24	11	13	14	19
IR	y	14	12	13	15	23	11	13	13	19
IR	y	14	12	14	16	24	11	13	13	19
IR	y	14	12	14	16	24	11	14	13	19
IR	y	14	12	13	16	24	11	13	13	20
IR	y	14	12	13	17	24	11	13	13	19
IR	y	14	12	13	16	25	12	14	13	18
IR	y	14	12	13	16	24	10	13	13	19
IR	y	14	12	13	16	24	11	13	13	19
IR	y	14	12	13	16	24	10	13	13	19
IR	y	14	12	13	16	24	10	13	13	21
IR	y	14	12	12	16	24	10	13	13	18
IR	y	14	12	14	16	24	11	13	13	19
IR	y	14	12	13	16	24	10	15	13	21
IR	y	14	12	14	16	24	11	13	13	21
IR	y	14	12	13	17	23	11	13	13	18
IR	y	14	12	14	16	24	11	13	13	20
IR	y	14	12	14	16	25	11	13	14	19
IR	y	14	12	12	17	24	10	13	13	19
IR	y	14	12	13	17	24	11	13	13	19
IR	y	15	12	13	16	24	11	13	13	18
IR	y	14	12	13	16	25	11	13	13	19
IR	y	14	12	13	16	24	11	13	13	19
IR	y	14	12	14	16	22	11	13	13	19
IR	y	14	12	13	16	24	11	13	13	18
IR	y	14	12	13	16	25	11	13	13	19
IR	y	14	12	14	17	24	11	13	13	20
IR	y	14	12	14	16	24	11	13	13	19
IR	y	14	12	14	16	24	11	13	13	20
IR	y	14	12	13	16	24	10	13	13	20
IR	y	14	12	13	16	24	11	13	13	20
IR	y	14	12	13	16	25	11	13	13	19
IR	y	14	12	13	16	24	11	13	13	19
IR	y	14	12	13	16	24	11	13	13	19
IR	y	14	12	13	17	24	11	13	13	18
IR	y	14	12	13	17	25	10	13	13	20
IR	y	14	12	13	16	24	11	13	12	19
IR	y	14	12	13	17	24	10	13	13	19
IR	y	14	12	13	16	24	12	13	13	19
IR	y	14	12	12	16	24	12	13	13	19
IR	y	14	12	13	16	24	10	13	13	18
IR	y	14	12	14	16	24	11	13	13	18
GE	y	14	12	12	15	25	10	13	13	19
GE	y	14	12	13	17	24	11	13	13	19
GE	y	15	12	13	16	24	10	13	14	19
GE	y	14	12	13	16	24	11	13	13	19
GE	y	15	12	13	16	24	11	13	13	19
GE	y	14	12	13	17	24	10	13	13	19
GE	y	14	12	13	15	24	11	13	13	18
GE	y	15	12	14	16	24	11	13	13	20
GE	y	14	13	13	16	24	11	13	12	20
GE	y	14	12	13	16	24	11	13	12	19
GE	y	14	12	13	16	24	11	13	14	18
GE	y	14	12	13	18	25	10	13	13	19
GE	y	14	12	14	16	24	11	13	13	19
GE	y	14	12	13	15	23	11	13	13	19
GE	y	14	12	14	16	24	11	13	13	19
GE	y	14	12	13	16	23	11	13	13	19
GE	y	14	12	13	16	25	10	13	13	19
GE	y	14	12	13	16	24	11	13	13	19
GE	y	14	12	13	16	23	11	13	13	19
GE	y	14	12	13	16	24	11	13	13	20
GE	y	15	12	13	16	24	10	13	13	19
GE	y	15	12	13	17	24	10	14	14	20
GE	y	14	12	14	16	24	11	13	13	19
GE	y	15	12	13	16	24	10	13	13	20
GE	y	14	12	13	16	24	10	13	13	19
GE	y	14	12	14	17	24	10	13	13	18
GE	y	14	12	12	16	23	10	13	13	20
GE	y	14	12	13	16	23	11	13	13	19
GE	y	14	12	13	16	24	11	13	13	19
GE	y	14	12	13	16	24	10	13	13	20
GE	y	14	12	13	15	23	11	13	13	19
GE	y	14	12	13	18	23	11	13	13	20
GE	y	14	12	13	16	24	11	13	13	19
GE	y	14	12	13	16	24	10	13	13	19
GE	y	14	12	13	15	23	10	13	13	18
GE	y	14	12	13	16	24	11	13	13	19
GE	y	14	12	14	16	24	10	13	13	19
GE	y	14	12	13	16	24	10	13	13	18
GE	y	15	12	13	17	23	11	15	13	18
GE	y	14	12	13	16	23	11	13	14	19
GE	y	14	12	13	17	24	11	12	13	18
GE	y	14	12	13	16	24	11	13	13	19
GE	y	14	12	13	16	24	11	13	13	19
GE	y	14	12	13	16	24	10	13	13	20

Population	All analyses?	DYS19	DYS388	DYS389I	DYS389b	DYS390	DYS391	DYS392	DYS393	DYS439
GE	y	14	12	13	15	23	10	13	13	18
GE	y	14	12	13	16	24	11	13	13	19
GE	y	14	12	13	15	23	10	13	13	21
GE	y	14	12	13	16	23	11	13	13	19
GE	y	14	12	13	16	24	11	13	13	20
GE	y	14	12	13	16	23	11	13	13	18
GE	y	14	12	13	17	25	10	13	13	19
GE	y	14	12	14	16	24	11	13	13	18
GE	y	15	12	13	16	23	10	13	13	19
GE	y	14	12	13	16	23	10	14	13	19
GE	y	13	12	13	16	23	11	13	13	18
GE	y	14	12	13	16	23	11	13	13	18
GE	y	14	12	13	16	24	11	13	13	19
GE	y	14	12	13	15	23	11	13	13	19
GE	y	14	12	13	16	23	11	12	13	20
GE	y	15	12	13	17	24	10	14	13	20
GE	y	14	12	13	16	24	11	14	13	19
GE	y	14	12	13	16	24	11	13	13	20
GE	y	15	12	13	17	23	11	13	13	19
GE	y	14	12	13	15	24	10	13	13	18
GE	y	14	12	13	16	24	11	13	13	20
GE	y	14	12	13	16	24	11	13	13	19
GE	y	14	12	14	16	24	11	13	13	20
GE	y	14	12	13	16	24	9	13	13	21
GE	y	14	12	13	16	25	11	13	12	21
GE	y	14	12	13	16	23	11	13	12	19
GE	y	14	12	12	16	24	10	13	13	19
GE	y	14	12	13	15	24	11	14	12	18
GE	y	14	11	13	16	25	11	13	13	19
GE	y	14	12	13	16	24	10	14	13	20
GE	y	14	12	13	17	24	11	13	13	19
FR4	y	14	12	13	16	24	11	13	13	18
FR4	y	14	12	13	17	24	11	13	13	18
FR4	y	14	12	13	16	24	11	13	14	18
FR4	y	14	12	13	17	24	11	13	13	18
FR4	y	14	12	13	16	24	11	13	13	19
FR4	y	14	12	13	16	24	11	13	13	19
FR4	y	14	12	14	16	24	11	13	13	18
FR4	y	14	12	13	16	24	12	13	13	18
FR4	y	14	12	13	17	24	11	13	13	19
FR4	y	14	12	14	16	25	11	13	13	19
FR4	y	14	12	13	16	23	10	13	13	18
FR4	y	15	12	13	16	25	11	13	13	18
FR4	y	14	12	12	17	24	10	13	13	19
FR4	y	15	12	13	16	25	11	13	13	19
FR4	y	14	11	13	16	24	11	14	14	18
FR4	y	15	12	13	17	24	10	13	13	18
FR4	y	14	12	12	17	24	11	13	13	19
FR4	y	14	13	13	15	24	11	13	13	20
FR4	y	14	12	13	16	24	12	13	13	18
FR4	y	14	12	14	16	24	11	13	13	19
FR4	y	14	12	12	16	24	11	13	13	18
FR4	y	14	12	13	16	23	11	13	13	18
FR4	y	14	12	13	16	24	11	13	13	20
FR4	y	14	12	13	16	24	11	13	13	18
FR4	y	14	12	14	16	24	11	13	13	19
FR4	y	14	12	13	16	24	10	13	13	19
FR4	y	14	12	13	17	25	10	14	13	18
FR4	y	14	12	13	16	24	10	13	13	19
FR4	y	14	12	13	16	24	11	13	13	19
FR4	y	15	12	13	15	24	10	13	13	19
FR4	y	14	12	13	16	23	11	13	13	19
FR4	y	15	12	14	16	22	10	13	13	20
FR4	y	13	12	13	16	24	11	13	13	19
FR4	y	14	12	13	16	25	11	13	13	19
FR4	y	14	12	13	16	24	10	13	13	18
FR4	y	14	12	13	16	25	11	13	13	18
FR4	y	14	12	13	16	24	11	13	13	19
FR4	y	14	12	14	16	23	10	13	13	19
FR4	y	14	12	13	16	24	11	13	13	20
FR4	y	14	12	14	16	23	10	13	13	20
FR4	y	15	12	13	16	24	11	13	13	18
FR4	y	14	12	13	17	24	12	13	13	20
FR4	y	14	12	13	16	23	11	13	13	19
FR4	y	14	12	13	17	24	11	13	13	19
SP5	y	14	12	13	16	25	11	13	13	18
SP5	y	14	12	13	16	24	10	13	13	19
SP5	y	13	12	14	16	24	13	13	14	18
SP5	y	14	12	12	16	23	11	13	13	19
SP5	y	14	12	14	17	24	11	13	13	20
SP5	y	14	12	13	15	24	11	13	13	20
SP5	y	14	12	14	18	24	11	13	13	20
SP5	y	14	12	14	16	23	10	13	13	20
SP5	y	14	12	14	16	24	10	12	13	20
SP5	y	14	12	13	15	24	11	13	13	20
SP5	y	14	12	14	16	23	10	13	13	20
SP5	y	14	12	14	17	24	11	12	13	20
SP5	y	14	12	13	16	24	10	13	13	19
SP5	y	14	12	13	16	24	10	13	12	19
SP5	y	14	12	12	16	24	10	14	13	19
SP5	y	15	12	14	16	24	10	13	13	20
SP5	y	15	12	13	16	24	11	13	12	20
SP5	y	14	12	13	17	25	11	13	13	19
SP5	y	14	12	13	17	24	10	13	13	19
SP5	y	14	12	13	18	24	11	13	13	18
SP5	y	15	12	13	16	23	11	13	13	19
SP5	y	14	12	14	17	24	11	13	13	18
SP5	y	14	12	12	16	23	11	13	13	19

Population	All analyses?	DYS19	DYS388	DYS389I	DYS389b	DYS390	DYS391	DYS392	DYS393	DYS439
SP5	y	15	12	13	16	24	11	13	13	20
SP5	y	14	12	13	16	24	11	13	13	20
SP5	y	15	12	13	16	23	11	13	13	19
SP5	y	14	12	14	16	24	11	13	13	19
SP5	y	15	12	13	16	24	11	15	13	18
SP5	y	15	12	12	16	24	11	13	13	19
SP5	y	14	12	13	16	24	11	13	13	20
SP5	y	15	12	13	16	23	11	13	13	20
SP5	y	14	12	13	16	24	11	13	13	18
SP5	y	14	12	13	16	25	11	13	14	19
SP5	y	14	12	13	16	24	11	13	13	20
SP5	y	14	12	13	16	23	10	13	13	19
SP5	y	14	12	13	16	23	11	13	13	19
SP5	y	14	12	14	16	24	10	13	13	19
SP5	y	14	12	14	16	24	10	13	13	19
SP5	y	14	12	14	16	24	12	13	13	19
SP5	y	14	12	13	16	24	10	13	13	20
SP5	y	14	12	13	17	24	11	13	12	19
SP5	y	14	12	14	16	23	11	13	13	20
SP5	y	14	12	13	16	24	11	13	13	19
SP5	y	14	12	13	17	25	11	13	13	19
SP5	y	14	12	13	16	24	11	13	13	18
SP4	y	14	12	13	16	24	10	13	13	20
SP4	y	15	12	13	16	24	10	13	13	20
SP4	y	14	12	13	16	24	11	13	13	18
SP4	y	14	12	14	16	24	11	13	13	18
SP4	y	14	12	13	16	24	11	13	13	19
SP4	y	14	13	13	16	24	11	13	13	19
SP4	y	14	12	13	16	24	11	13	13	20
SP4	y	14	12	13	16	24	11	13	13	19
SP4	y	15	12	13	16	24	10	13	13	18
SP4	y	14	12	13	16	24	12	14	14	19
SP4	y	14	12	14	16	24	11	13	13	20
SP4	y	14	12	13	16	23	11	14	14	19
SP4	y	14	12	13	17	24	10	13	13	18
SP4	y	14	12	13	16	24	11	12	12	19
SP4	y	14	12	13	16	23	11	13	13	19
SP4	y	14	12	14	17	24	11	12	13	19
SP4	y	14	12	12	16	26	10	13	13	19
SP4	y	14	12	14	17	24	11	13	13	18
SP4	y	14	12	13	16	25	11	13	13	19
SP4	y	14	12	13	17	24	10	14	13	19
SP4	y	14	12	13	17	24	10	13	13	19
SP4	y	14	12	13	16	24	11	13	13	19
SP4	y	14	12	14	16	24	12	13	13	19
SP4	y	14	12	14	16	24	11	13	13	19
SP4	y	14	14	14	16	23	11	13	13	19
SP4	y	14	12	13	16	24	10	13	13	19
SP4	y	14	12	13	17	24	11	13	12	19
SP4	y	14	12	14	16	24	11	13	14	18
SP4	y	14	12	14	16	24	11	13	13	18
SP4	y	14	12	13	16	24	11	13	12	19
SP4	y	14	12	13	16	25	10	13	12	19
SP4	y	15	12	14	17	24	11	13	12	19
SP4	y	14	12	13	16	24	10	13	13	20
SP4	y	15	12	14	16	24	11	13	13	19
SP4	y	14	12	12	16	24	11	13	13	19
SP4	y	14	12	12	16	25	11	13	13	19
SP4	y	14	12	13	18	25	11	13	13	19
SP4	y	12	12	13	17	24	10	13	13	20
SP4	y	15	12	13	16	23	10	13	13	19
SP4	y	14	12	13	16	24	11	13	12	19
SP4	y	14	12	13	16	25	10	13	13	19
SP4	y	14	12	13	16	24	11	13	13	20
SP4	y	14	12	12	16	25	11	13	13	19
SP4	y	14	12	14	16	24	10	13	13	18
SP4	y	14	12	13	17	24	10	13	13	19
SP4	y	14	12	13	16	23	10	13	13	18
SP4	y	14	12	14	16	24	10	13	13	19
SP4	y	14	12	13	17	23	11	13	13	18
SP4	y	14	12	13	16	24	11	13	13	19
SP4	y	14	12	13	16	24	10	13	13	18
SP4	y	14	12	13	16	24	11	13	13	19
SP4	y	14	12	13	16	24	10	13	13	19
SP4	y	15	12	13	17	24	11	13	13	19
SP4	y	14	12	13	16	24	10	13	14	19
SP4	y	14	12	13	18	24	12	13	13	19
SP4	y	14	12	13	16	24	11	13	13	20
SP4	y	14	12	13	16	24	11	12	13	19
SP4	y	14	12	13	16	24	10	13	13	19
SP4	y	14	12	13	16	24	11	12	14	18
SP4	y	14	12	12	16	24	11	13	13	20
SP4	y	14	12	13	16	24	11	13	13	19
SP4	y	14	12	13	16	23	11	13	13	21
SP4	y	14	12	13	16	25	11	13	13	19
SP4	y	14	12	14	16	24	11	13	13	18
SP4	y	14	12	13	16	24	11	13	13	18
SP6	y	14	12	13	16	25	11	13	13	19
SP6	y	14	12	13	16	23	10	13	13	19
SP6	y	14	12	13	16	24	11	13	12	19
SP6	y	14	12	13	16	24	11	13	13	19
SP6	y	14	12	13	16	24	11	13	13	18
SP6	y	14	12	13	16	24	11	14	13	19
SP6	y	14	12	12	16	23	11	13	13	18
SP6	y	14	12	13	16	25	11	14	13	19
SP6	y	14	12	13	14	24	11	13	14	19
SP6	y	14	12	13	17	23	10	13	13	20
SP6	y	15	12	13	16	24	10	13	13	19

Population	All analyses?	DYS19	DYS388	DYS389I	DYS389b	DYS390	DYS391	DYS392	DYS393	DYS439
SP6	y	14	12	13	16	24	11	14	14	19
SP6	y	13	12	13	16	25	11	13	13	20
SP6	y	14	12	13	15	23	12	13	13	18
SP6	y	14	12	13	16	24	11	13	13	20
SP6	y	14	12	13	17	24	11	13	13	19
SP6	y	14	12	13	16	24	11	13	13	18
SP6	y	14	12	13	16	24	11	13	12	19
SP6	y	14	12	13	14	24	11	13	13	20
SP6	y	14	12	13	16	24	11	13	15	19
SP6	y	14	12	14	15	24	11	13	13	20
SP6	y	15	12	13	16	24	10	13	14	18
SP6	y	14	12	12	15	24	10	13	13	18
SP6	y	14	12	13	16	24	10	13	13	20
SP6	y	14	12	13	16	24	11	13	13	20
SP6	y	15	12	13	16	24	11	13	13	19
SP6	y	14	12	12	16	24	10	13	13	19
SP6	y	14	12	13	16	24	11	13	13	19
SP6	y	15	12	13	16	24	10	13	13	19
SP6	y	14	12	13	16	23	12	13	13	20
SP6	y	14	12	14	17	24	10	13	14	21
SP6	y	14	12	13	16	24	10	13	13	19
SP6	y	14	12	15	16	24	11	13	13	21
SP6	y	14	12	14	16	23	11	13	13	19
SP6	y	14	12	13	17	24	11	14	13	20
SP6	y	14	12	13	16	24	10	13	13	18
SP6	y	14	12	14	17	23	11	13	13	20
SP6	y	14	12	13	16	23	10	13	13	19
SP6	y	14	12	13	16	24	11	14	13	19
SP6	y	14	12	14	16	24	11	13	13	19
SP6	y	14	12	12	16	24	11	13	12	19
SP6	y	14	12	13	15	22	10	13	13	18
SP6	y	14	12	13	16	25	11	14	13	19
SP6	y	14	12	13	15	25	10	13	14	20
SP6	y	15	12	13	16	24	11	13	13	19
SP6	y	14	12	13	17	24	11	13	13	19
SP6	y	14	12	13	16	25	11	13	13	18
SP6	y	14	12	12	16	24	11	13	13	20
SP6	y	14	12	13	16	24	11	13	13	18
SP6	y	14	12	13	16	23	11	13	13	19
SP2	y	14	12	13	16	24	10	13	13	19
SP2	y	14	12	13	16	24	11	13	13	19
SP2	y	14	12	13	16	25	10	13	13	20
SP2	y	14	12	12	16	23	11	14	13	18
SP2	y	14	12	13	16	24	11	13	13	19
SP2	y	14	12	14	16	24	11	13	12	20
SP2	y	14	12	12	16	24	11	13	13	19
SP2	y	14	12	14	16	24	11	13	13	18
SP2	y	14	12	13	16	24	11	13	13	19
SP2	y	14	12	13	16	25	10	13	13	20
SP2	y	14	13	12	16	24	10	13	13	19
SP2	y	14	12	13	16	24	11	13	13	19
SP2	y	14	12	13	17	25	11	13	13	19
SP2	y	15	12	13	16	23	11	13	13	19
SP2	y	14	12	13	16	24	10	13	13	19
SP2	y	14	12	13	16	24	11	13	13	19
SP2	y	14	12	14	17	24	10	13	13	20
SP2	y	14	12	13	16	24	11	13	12	19
SP2	y	14	12	13	16	23	10	13	13	19
SP2	y	15	12	13	16	24	11	13	13	21
SP2	y	14	12	14	17	25	10	13	13	19
SP2	y	14	12	13	16	24	10	13	13	19
SP2	y	14	12	14	15	24	10	13	13	19
SP2	y	15	12	13	16	24	10	13	13	19
SP2	y	14	12	13	16	24	11	14	13	19
SP2	y	14	12	13	16	24	10	13	13	19
SP2	y	14	12	13	18	24	10	13	14	19
SP2	y	14	12	12	16	24	11	13	13	19
SP2	y	14	12	13	16	24	11	13	13	20
SP2	y	14	12	12	16	23	11	13	14	19
SP2	y	14	12	14	18	24	11	13	14	18
SP2	y	14	12	13	16	24	11	13	13	19
SP2	y	14	12	13	16	23	10	13	13	20
SP2	y	14	12	13	17	23	10	13	13	19
SP2	y	14	12	12	16	24	11	13	13	20
SP2	y	14	12	14	17	24	10	13	13	19
SP2	y	14	12	13	15	24	11	13	13	18
SP2	y	14	12	13	16	25	11	13	13	18
SP2	y	14	12	13	16	23	10	13	13	18
TK3	y	14	12	13	16	24	11	13	12	20
TK3	y	14	12	13	16	24	11	13	12	19
TK3	y	14	12	13	16	24	12	13	13	19
TK3	y	14	12	13	16	25	11	14	13	18
TK3	y	14	12	13	15	24	11	15	12	20
TK3	y	15	12	13	16	24	11	13	12	19
TK3	y	14	12	13	16	21	10	13	12	19
TK3	y	14	12	13	16	24	11	13	13	19
TK3	y	13	12	13	17	25	11	13	12	19
TK3	y	14	12	13	16	23	10	13	12	19
TK3	y	14	12	13	18	24	10	13	12	18
TK3	y	14	12	12	16	24	11	13	14	18
TK3	y	14	12	13	16	24	11	13	13	18
TK3	y	14	12	13	15	24	11	14	12	19
TK3	y	14	12	13	16	24	11	15	13	19
TK3	y	14	12	13	15	24	11	14	12	20
TK3	y	13	12	13	18	25	11	13	12	18
TK3	y	14	12	13	16	25	11	13	12	19
TK3	y	14	12	13	15	23	10	14	12	19
TK3	y	14	12	13	16	24	11	13	13	19

Population	All analyses?	DYS19	DYS388	DYS389I	DYS389b	DYS390	DYS391	DYS392	DYS393	DYS439
TK3	y	14	13	14	16	23	10	13	12	18
TK3	y	14	12	13	15	24	11	14	12	19
TK1	y	14	12	14	16	24	10	13	12	20
TK1	y	14	12	13	15	23	11	13	13	19
TK1	y	14	12	13	15	24	10	14	12	20
TK1	y	14	12	13	16	24	10	13	12	20
TK1	y	14	12	12	16	25	11	13	12	20
TK1	y	14	12	14	17	25	10	13	12	19
TK1	y	14	12	13	16	24	11	13	12	20
TK1	y	14	12	14	17	23	10	13	12	19
TK1	y	14	12	14	16	24	11	13	13	18
TK1	y	14	12	13	16	24	11	13	12	19
TK1	y	14	12	14	16	24	11	13	13	18
TK1	y	14	12	13	16	24	11	13	13	19
TK1	y	14	12	13	16	25	11	13	12	18
TK1	y	15	12	12	17	24	10	13	13	20
TK1	y	15	12	14	15	25	10	12	12	18
TK1	y	14	12	13	16	24	11	13	12	19
TK1	y	15	12	13	16	21	10	13	12	19
TK1	y	14	12	13	17	25	10	13	12	19
TK1	y	14	12	13	16	24	11	13	14	19
TK1	y	15	12	12	15	23	10	14	12	19
TK1	y	14	12	13	16	24	11	13	12	19
TK1	y	14	12	14	16	24	11	13	12	18
TK1	y	14	12	14	16	24	11	12	12	18
TK1	y	14	12	13	16	24	10	14	12	20
TK1	y	15	12	13	16	23	11	13	12	19
TK1	y	14	12	13	16	24	10	12	12	19
TK1	y	14	12	13	16	23	10	13	12	19
TK1	y	14	12	13	15	24	10	14	12	18
TK1	y	14	12	13	15	24	11	14	12	20
TK2	y	14	12	13	16	25	10	13	12	20
TK2	y	14	12	13	15	24	10	15	12	19
TK2	y	14	12	13	16	24	10	13	12	19
TK2	y	14	12	12	15	24	10	14	12	19
TK2	y	13	12	13	17	24	11	13	12	21
TK2	y	14	12	12	16	24	11	13	13	21
TK2	y	14	12	12	16	24	11	13	13	21
TK2	y	14	12	13	16	23	11	13	12	18
TK2	y	14	12	13	16	24	11	13	13	19
TK2	y	14	12	13	16	24	11	13	12	19
TK2	y	13	12	13	16	25	11	13	12	19
TK2	y	14	12	13	16	24	11	13	12	19
TK2	y	14	12	13	16	24	11	13	12	20
TK2	y	14	12	13	16	24	11	13	12	20
TK2	y	14	12	13	16	24	11	13	12	20
TK2	y	14	12	13	16	24	11	13	12	18
TK2	y	14	12	13	16	24	11	13	12	19
TK2	y	14	12	13	15	23	11	11	12	19
TK2	y	14	12	13	16	24	10	13	12	19
TK2	y	15	12	12	15	24	10	13	12	18
TK2	y	14	12	13	16	25	11	13	12	18
TK2	y	14	12	12	15	24	10	14	12	19
TK2	y	14	12	13	16	25	11	13	12	18
TK2	y	14	12	12	15	24	10	14	12	19
TK2	y	14	12	13	16	24	10	13	12	19
SB		14	12	13	16	24	11	13	13	20
SB		14	12	13	16	24	11	13	12	19
SB		14	12	13	16	24	11	13	13	19
SB		15	12	13	16	24	11	13	12	19
SB		14	12	13	16	24	10	13	13	18
SB		14	12	13	16	24	11	13	13	19
SB		14	12	13	16	23	11	13	13	21
GR		15	12	13	16	24	11	13	13	17
GR		14	12	13	16	23	11	14	13	19

Population abbreviations are as in Table 1; for each microsatellite (DYS19 - DYS439) repeat unit numbers are given.
