# Supplementary material for: A Predominantly Neolithic Origin for European Paternal Lineages
Source: PLoS Biol. 2010 Jan 19;8(1):e1000285. doi: 10.1371/journal.pbio.1000285 (PMC2799514; doi:10.1371/journal.pbio.1000285)
Supplement: Text S1 — Details of application of BATWING. (0.14 MB DOC) [file pbio.1000285.s002.doc]

**Text S1: Details of application of BATWING**

TMRCA, population growth rates, and the times of individual population splits were estimated using BATWING^1^, under a model of exponential population growth and splitting. Standard use of BATWING assumes a random sample from a population, rather than a random sample of individuals sharing a haplogroup. Wiuf^2^ showed that expected coalescence times for a subsample that all share a rare allele (equivalent to a haplogroup) are approximately proportional to a sample from the coalescent, with a scaling equal to the frequency of the rare allele in the population. Other simulations performed by us (not shown) demonstrate that common haplogroups have mean coalescence times that follow the same approximation, provided sample sizes for the haplogroup are large (>100). Simulation studies of the bias and residual mean square error of point estimates (Table 1 below) show that BATWING analyses based only on haplogroups, while underperforming random samples of the same size, give realistic estimates of important parameters, and the time to the most recent common ancestor of the haplogroup. More stringent tests of the accuracy of the posterior density estimates are summarised in Table 2, below. The coverage is approximately correct for the haplogroup data-sets for the correct priors; incorrect priors gave coverages that overestimated (for TMRCA) and underestimated (for α and N) the posterior uncertainty. Note that there is a tendency for TMRCA to be underestimated when single-haplogroup data are considered.

In choosing a microsatellite mutation rate there are two approaches to dealing with recurrent mutation and drift: one is to average over a long time period, yielding the 'effective' mutation rate^3^, and the other is to model mutation rates explicitly for each locus, using information from known pedigrees^1^. In our analyses of hg R1b1b2 data we used an individual mutation rate prior for each marker, calculated from multiple pedigrees^4-15^, where the parameters of the gamma distribution represent, respectively, number of observed mutations and number of meioses: gamma(22,8856) for DYS19, gamma(1,2135) for DYS388, gamma(17,7273) for DYS389I, gamma(19,7260) for DYS389b, gamma(19,7949) for DYS390, gamma(26,7897) for DYS391, gamma(4,6094) for DYS392, gamma(6,6651) for DYS393 and gamma(27,4205) for DYS439. For the initial population size, we used three broad gamma priors with both means and standard deviations of 10000, 5000 and 3000 respectively. For population growth rate per generation, we also used the broad prior distributions gamma(2,400) (mean = 0.005, SD = 0.0035) or gamma(1,200) (mean = 0.005, SD = 0.005). Since we are considering not an entire population, but a single lineage, the population size N is scaled by the overall frequency of the lineage in the dataset (~0.50). The TMRCA is calculated using the product of the estimated population size N and the height of the tree T (any scaling factors cancel). A generation time of 30 years^16^ was used to produce a time estimate in years. A pair of runs of four million MCMC cycles were used, with 100 attempted tree changes within each MCMC cycle. The first 1.6 million iterations were discarded as burn-in and samples taken every 200 cycles to provide 40,000 MCMC cycles for inference. Convergence was checked by comparing parallel runs and by analysing autocorrelations within runs. All statistics were calculated in the R statistics package^17^.

| f | prior |  | Single-haplogroup Data | | All-haplogroup Data | | Sub-sampled all-haplogroup Data | |
| --- | --- | --- | --- | --- | --- | --- | --- | --- |
|  |  |  | Bias | RMSE | Bias | RMSE | Bias | RMSE |
|  |  | TMRCA | 0.01 | 0.22 | -0.07 | 0.22 | -0.09 | 0.24 |
| 0.2 | A | α | 0.79 | 0.86 | 0.06 | 0.23 | 0.03 | 0.24 |
|  |  | N | 0.11 | 0.19 | 0.00 | 0.04 | 0.00 | 0.06 |
|  |  | TMRCA | -0.02 | 0.1 | -0.03 | 0.05 | -0.02 | 0.06 |
|  | B | α | 0.48 | 3.08 | 0.00 | 0.81 | 0.27 | 1.96 |
|  |  | N | 13.11 | 14.38 | 1.66 | 2.06 | 2 | 2.65 |
|  |  | TMRCA | -0.13 | 0.29 | -0.21 | 0.35 | -0.22 | 0.36 |
| 0.5 | A | α | 0.45 | 0.55 | -0.02 | 0.31 | -0.04 | 0.32 |
|  |  | N | 0.07 | 0.11 | 0.00 | 0.04 | 0.00 | 0.04 |
|  |  | TMRCA | -0.01 | 0.07 | -0.03 | 0.05 | -0.02 | 0.07 |
|  | B | α | 0.53 | 2.08 | 0.00 | 1.08 | 0.13 | 1.62 |
|  |  | N | 6.14 | 6.71 | 1.68 | 2.2 | 1.76 | 2.44 |

**Table 1**. Bias and root mean squared error (RMSE) of point estimates for TMRCA (in thousands of generations), growth rate α (as a percentage per generation) and N (in thousands of males) based on the median of the posterior distribution for simulated single-haplogroup data-set, all-haplogroup data-set and sub-sampled all-haplogroup data-set for tight (A) and diffuse (B) priors, for frequency of haplogroup of 0.2 and 0.5. Size of single-haplogroup sample and sub-sampled all-haplogroup sample are 100, full all-haplogroup data-sets have sizes 200 (for f=0.5) and 500 (for f=0.2). All values based on 1000 simulated data-sets. BATWING output is based on 20,000 samples thinned from output of length 3.2×10^7^ after burn in of 1.2×10^6^ iterations.

| f | prior |  | Single-haplogroup Data | | | All-haplogroup Data | | | Sub-sampled all-haplogroup Data | | |
| --- | --- | --- | --- | --- | --- | --- | --- | --- | --- | --- | --- |
|  |  |  | 50% | 80% | 90% | 50% | 80% | 90% | 50% | 80% | 90% |
|  |  | TMRCA | 0.40 | 0.67 | 0.81 | 0.53 | 0.80 | 0.90 | 0.51 | 0.81 | 0.90 |
| 0.2 | A | α | 0.47 | 0.78 | 0.88 | 0.47 | 0.74 | 0.86 | 0.47 | 0.77 | 0.86 |
|  |  | N | 0.44 | 0.72 | 0.83 | 0.49 | 0.78 | 0.89 | 0.47 | 0.78 | 0.90 |
|  |  | TMRCA | 0.67 | 0.93 | 0.97 | 0.79 | 0.98 | 0.99 | 0.84 | 0.99 | 1.00 |
|  | B | α | 0.16 | 0.76 | 0.96 | 0.87 | 1.00 | 1.00 | 0.88 | 1.00 | 1.00 |
|  |  | N | 0.07 | 0.65 | 0.89 | 0.89 | 1.00 | 1.00 | 0.98 | 1.00 | 1.00 |
|  |  | TMRCA | 0.35 | 0.58 | 0.71 | 0.51 | 0.80 | 0.88 | 0.49 | 0.79 | 0.88 |
| 0.5 | A | α | 0.37 | 0.65 | 0.78 | 0.34 | 0.58 | 0.68 | 0.35 | 0.58 | 0.68 |
|  |  | N | 0.43 | 0.71 | 0.82 | 0.50 | 0.81 | 0.90 | 0.50 | 0.77 | 0.88 |
|  |  | TMRCA | 0.80 | 0.98 | 1.00 | 0.81 | 0.98 | 1.00 | 0.83 | 0.99 | 1.00 |
|  | B | α | 0.67 | 0.99 | 1.00 | 0.92 | 0.99 | 1.00 | 0.91 | 1.00 | 1.00 |
|  |  | N | 0.38 | 0.94 | 0.99 | 0.93 | 1.00 | 1.00 | 0.95 | 1.00 | 1.00 |

**Table 2**. Posterior coverage of true values for TMRCA, α and N for simulated haplotype data-set, full data-set and sub-sampled data-set for tight (A) and diffuse (B) priors, for frequency of haplogroup f of 0.2 and 0.5. Size of single-haplogroup sample and sub-sampled all-haplogroup data-sets are 100, full all-haplogroup data-sets have sizes 200 (for f=0.5) and 500 (for f=0.2). The values represent the proportion of times the true value lies within the 50%, 80% and 90% equal-tail posterior probability interval for these parameters. All values based on 1000 simulated data-sets. Maximum standard errors on these values are 0.016. BATWING output is based on 20,000 samples thinned from output of length 3.2×10^7^ after burn in of 1.2×10^6^ iterations.

**References**

1. Wilson, I. J., Weale, M. E., and Balding, D. J., Inferences from DNA data: population histories, evolutionary processes and forensic match probabilities. *J. Roy. Statist. Soc. A* **166**, 1-33 (2003).

2. Wiuf, C., On the genealogy of a sample of neutral rare alleles. *Theor. Popul. Biol.* **58**, 61-75 (2000).

3. Zhivotovsky, L. A. et al., The effective mutation rate at Y chromosome short tandem repeats, with application to human population-divergence time. *Am. J. Hum. Genet.* **74**, 50-61 (2004).

4. Bianchi, N.O. et al., Characterization of ancestral and derived Y-chromosome haplotypes of new world native populations. *Am. J. Hum. Genet.* **63**, 1862-1871 (1998).

5. Kayser, M. et al., Characteristics and frequency of germline mutations at microsatellite loci from the human Y chromosome, as revealed by direct observation in father/son pairs. *Am. J. Hum. Genet.* **66**, 1580-1588 (2000).

6. Kurihara, R. et al., Mutations in 14 Y-STR loci among Japanese father-son haplotypes. *Int. J. Legal. Med.* **118**, 125-31 (2004).

7. Dupuy, B. M., Stenersen, M., Egeland, T., and Olaisen, B., Y-chromosomal microsatellite mutation rates: differences in mutation rate between and within loci. *Hum. Mutat.* **23**, 117-24 (2004).

8. Budowle, B. et al., Twelve short tandem repeat loci Y chromosome haplotypes: genetic analysis on populations residing in North America. *Forensic Sci. Int.* **150**, 1-15 (2005).

9. Ballard, D. J. et al., A study of mutation rates and the characterisation of intermediate, null and duplicated alleles for 13 Y chromosome STRs. *Forensic Sci. Int.* **155**, 65-70 (2005).

10. de Souza Goes, A. C. et al., Population and mutation analysis of 17 Y-STR loci from Rio de Janeiro (Brazil). *Int. J. Legal Med.* **119**, 70-6 (2005).

11. Turrina, S., Atzei, R., and De Leo, D., Y-chromosomal STR haplotypes in a Northeast Italian population sample using 17plex loci PCR assay. *Int. J. Legal Med.* **120**, 56-59 (2006).

12. Lee, H. Y. et al., Haplotypes and mutation analysis of 22 Y-chromosomal STRs in Korean father-son pairs. *Int. J. Legal Med.* **121**, 128-135 (2007).

13. Hohoff, C. et al., Y-chromosomal microsatellite mutation rates in a population sample from northwestern Germany. *Int. J. Legal Med.* **121**, 359-63 (2007).

14. Domingues, P. M. et al., Sub-Saharan Africa descendents in Rio de Janeiro (Brazil): population and mutational data for 12 Y-STR loci. *Int. J. Legal Med.* **121**, 238-41 (2007).

15. Decker, A.E. et al., Analysis of mutations in father–son pairs with 17 Y-STR loci. *Forensic Sci. Internat. Genet.* **2**, e31-e35 (2008).

16. Fenner, J. N., Cross-cultural estimation of the human generation interval for use in genetics-based population divergence studies. *Am. J. Phys. Anthropol.* **128**, 415-423 (2005).

17. R Development Core Team, *R: A language and environment for statistical computing*. (R Foundation for Statistical Computing, Vienna, Austria, 2007).
